# Supplementary material for: Structural basis of tRNA recognition by the widespread OB fold
Source: Nat Commun. 2024 Jul 29;15:6385. doi: 10.1038/s41467-024-50730-1 (PMC11286949; doi:10.1038/s41467-024-50730-1)
Supplement: Supplementary file 1 — Supplementary Information [file 41467_2024_50730_MOESM1_ESM.pdf]

Supplementary Information for:

Structural basis of tRNA recognition by the widespread OB fold

Aline Umuhire Juru, Rodolfo Ghirlando and Jinwei Zhang

Supplementary Tables 1-5.
Supplementary Figs. 1-14.

Supplementary Table 1. **| X-ray crystallography statistics for free *Aquifex aeolicus* Trbp111 and Trbp111-tRNA complex crystals**

Crystals	*Aquifex aeolicus* Trbp111	Trbp111-tRNA^{Ile} complex
**Data collection**		
Wavelength (Å)	1.000	1.000
Space group	$C\ 2\ 2\ 2_1$	$C\ 2_1$
Cell dimensions		
a, b, c (Å)	58.0, 82.2, 88.9	190.5, 55.0, 87.2
α, β, γ (°)	90, 90, 90	90, 94.4, 90
Resolution (Å)[a]	47.4 - 2.3 (2.383 - 2.3)	43.09 - 2.64 (2.734 - 2.64)
R_{merge} (%)[a]	0.1775 (0.4027)	0.1491 (0.6814)
$\langle I\rangle/\langle\sigma(I)\rangle$[a]	9.53 (1.79)	9.76 (0.87)
Completeness (%)[a]	99.70 (98.10)	98.79 (99.29)
Multiplicity[a]	18.6 (11.7)	12.6 (13.1)
$CC_{1/2}$	0.996 (0.954)	0.977 (0.909)
CC^*	0.999 (0.988	0.994 (0.976)
**Refinement**		
Resolution (Å)[a]	47.4 - 2.3 (2.383 - 2.3)	43.09 - 2.64 (2.734 - 2.64)
No. reflections[b]	180429 (10898)	333857 (34552)
R_{work}/ R_{free} (%)[a]	21.42 (25.58) / 26.41 (31.94)	18.27 (38.39) / 22.17 (39.30)
No. atoms	1779	5091
Macromolecules	1683	4890
Ligand/ion	5	0
Water	91	201
Mean B-factors (Å^2)	33.4	57.49
Macromolecules	33.39	52.38
Ligand/ion	36.23	
Water	33.26	50.38
R.m.s. deviations		
Bond lengths (Å)	0.002	0.003
Bond angles (°)	0.51	0.74
Maximum likelihood coordinate precision (Å)	0.31	0.47
PDB accession code	8VTZ	8VU0

[a] Values in parentheses are for the highest resolution shell. [b] Values in parentheses are for the cross-validation set.

Supplementary Table 2. | Effects of Trbp111 and Arc1pΔN on 2AP fluorescence lifetimes. Individual, deconvoluted 2AP fluorescence lifetimes (τ_1 - τ_3) and corresponding amplitudes (A1 – A3), from three-component fits of representative measurements. Average lifetimes (τ_{avg}) are amplitude-weighted lifetime averages ± s.d. from n = 3 biologically independent replicates. MiniH: minihelix. Only the sequences of the 3′-most four nucleotides are shown. See also Supplementary Fig. 8.

**Sample**	**τ_1 (ns)**	**A1**	**τ_2 (ns)**	**A2**	**τ_3 (ns)**	**A3**	**τ_{avg} (ns)**	**χ^2**
**MiniH-ACC-2AP with Trbp111**								
RNA only	0.50	0.04	4.34	0.42	6.77	0.53	5.48 ± 0.03	1.03
WT 20 μM	0.48	0.04	4.04	0.35	6.83	0.61	5.62 ± 0.03	1.00
WT 60 μM	0.62	0.04	4.50	0.51	7.72	0.45	5.78 ± 0.02	1.05
R75A 20 μM	0.55	0.05	4.58	0.50	7.03	0.46	5.49 ± 0.01	1.05
R75A 60 μM	0.55	0.04	4.22	0.37	6.80	0.58	5.55 ± 0.02	0.98
**MiniH-AC-2AP-A with Trbp111**								
RNA only	0.73	0.18	3.09	0.39	7.84	0.43	4.73 ± 0.02	1.02
WT 20 μM	0.74	0.16	3.15	0.37	8.10	0.47	5.09 ± 0.03	1.06
WT 60 μM	0.74	0.13	3.38	0.36	8.80	0.51	5.76 ± 0.01	0.99
R75A 20 μM	0.72	0.17	3.08	0.39	7.84	0.44	4.75 ± 0.01	1.08
R75A 60 μM	0.77	0.17	3.18	0.39	8.18	0.43	4.93 ± 0.01	1.04
**MiniH-ACC-2AP with Arc1p**								
RNA only	0.49	0.04	4.05	0.30	6.53	0.66	5.46 ± 0.07	1.02
ΔN 20 μM	0.60	0.04	4.13	0.38	8.13	0.58	6.26 ± 0.04	0.96
ΔN-K269A 20 μM	0.61	0.05	4.45	0.47	7.39	0.48	5.70 ± 0.01	1.00
**MiniH-AC-2AP-A with Arc1p**								
RNA only	0.72	0.18	3.04	0.35	7.71	0.47	4.76 ± 0.03	1.08
ΔN 20 μM	0.72	0.09	3.39	0.31	8.45	0.60	6.2 ± 0.02	1.04
ΔN-K269A 20 μM	0.76	0.16	3.37	0.36	8.38	0.48	5.34 ± 0.04	1.09

Supplementary Table 3 | Thermodynamic parameters from ITC analysis of WT *E. coli* $tRNA^{Ile}$ binding to different Arc1p constructs.

Data are mean ± s.d. from n biologically independent replicates. n = 4 for WT ΔN, ΔN R274A; n = 3 for Arc1p (full-length), ΔN S278A, ΔN M281A, ΔN S278A/M281A, ΔN V271A/M273A/M281A, ΔN K269A.

Arc1p construct	K_d (nM)	ΔH (kcal mol^{-1})	$-T\Delta S$ (kcal mol^{-1})
**Arc1p (full-length)**	1280 ± 130	-11.11 ± 0.68	3.07 ± 0.73
**ΔN**	321 ± 101	-10.741 ± 1.07	1.86 ± 1.22
**ΔNΔM1**	2553 ± 456	-7.65 ± 0.03	0.023 ± 0.14
**ΔN-S278A**	845 ± 149	-7.215 ± 0.26	-1.08 ± 0.31
**ΔN-M281A**	384 ± 48	-8.83 ± 0.50	0.074 ± 0.44
**ΔN-S278A/M281A**	1433 ± 379	-7.43 ±0.54	-0.48 ± 0.55
**ΔN-K269A**	1497 ± 55	-10.33 ± 0.10	2.389 ± 0.12
**ΔN-V271A/M273A/M281A**	544 ± 215	-9.04 ± 0.28	0.46 ± 0.35
**ΔN-R274A**	2975 ± 674	-6.22 ± 0.71	-1.34 ± 0.70

Supplementary Table 4 | Thermodynamic parameters from ITC analysis of WT Arc1pΔN binding to various *E. coli* tRNA^{Ile} constructs.

Data are mean ± s.d. from n biologically independent replicate. n = 4 for WT, Δ3′A; n = 2 for ASL^{GAAA}, 3′UGGU, ASL^{GAAA}/5′GC, ASL^{GAAA}/5′GGCC, G18C/G19C/ΔACCA; n = 3 for ΔACCA, ASL^{GAAA}/ΔACCA; n = 9 for G18C/G19C.

**E. coli tRNA^{Ile}**	***K*$_d$ (nM)**	**Δ*H* (kcal mol^{-1})**	**-*T*Δ*S* (kcal mol^{-1})**
**WT**	321 ± 101	-10.74 ± 1.07	1.85875 ± 1.22
**Δ3′A**	1043 ± 295	-5.84 ± 0.82	-2.34 ± 0.91
**ASL^{GAAA}**	252 ± 110	-12.47 ± 0.25	3.44 ± 0.02
**ASL^{GAAA}/ΔACCA**	1746 ± 958	-6.9 ± 1.69	-1.03 ± 2.05
3′**UGGU**	654 ± 165	-8.22 ± 1.04	-0.228 ± 1.20
**ASL^{GAAA}/** 5′**GC**	1500 ± 283	-5.24 ± 0.11	-2.70 ± 0.01
**ASL^{GAAA}/** 5′**GGCC**	3300 ± 1980	-6.23 ± 1.06	-1.31 ± 1.45
**G18C/G19C**	3325 ± 1477	-10.62 ± 1.93	3.04 ± 1.90
**G18C/G19C/ΔACCA**	2785 ± 304	-2.89 ± 0.62	-4.68 ± 0.69
**ΔACCA**	2100 ± 985	-7.65 ± 2.98	-0.22 ± 3.43

Supplementary Table 5 | List of 18 AlphaFold-predicted, OB-fold-containing proteins in *Saccharomyces cerevisiae* identified by Foldseek[1].

Search was performed using *Aquifex aeolicus* Trbp111 (PDB: 1PYB)[2] against the AlphaFold/Proteome v4 database, in *Saccharomyces cerevisiae* proteome.

Target	Description	Prob.	E-Value	Score	Query Pos.	Target Pos.
AF-P46672-F1-model_v4	tRNA-aminoacylation cofactor ARC1	1	4.40E-05	217	12-105 (107)	210-305 (376)
AF-P53732-F1-model_v4	37S ribosomal protein S12, mitochondrial	0.25	8.37E-01	42	1-107 (107)	45-150 (153)
AF-Q02950-F1-model_v4	37S ribosomal protein MRP51, mitochondrial	0.16	1.12E+00	37	13-98 (107)	226-302 (344)
AF-P38861-F1-model_v4	60S ribosomal export protein NMD3	0.12	4.32E+00	33	12-101 (107)	314-408 (518)
AF-P04802-F1-model_v4	Aspartate--tRNA ligase, cytoplasmic	0.12	2.87E+00	33	1-95 (107)	94-198 (557)
AF-Q3E7X9-F1-model_v4	40S ribosomal protein S28-A	0.1	5.16E+00	31	10-69 (107)	4-61 (67)
AF-P33299-F1-model_v4	26S proteasome regulatory subunit 7 homolog	0.1	3.63E+00	31	2-83 (107)	84-187 (467)
AF-Q08162-F1-model_v4	Exosome complex exonuclease DIS3	0.1	3.63E+00	31	1-65 (107)	260-319 (1001)
AF-P24384-F1-model_v4	Pre-mRNA-splicing factor ATP-dependent RNA helicase PRP22	0.1	1.69E+00	31	1-107 (107)	153-261 (1145)
AF-P0CX30-F1-model_v4	40S ribosomal protein S23-B	0.09	1.59E+00	30	12-93 (107)	47-124 (145)
AF-Q01939-F1-model_v4	26S proteasome regulatory subunit 8 homolog	0.09	3.63E+00	30	5-83 (107)	58-126 (405)
AF-P0CX29-F1-model_v4	40S ribosomal protein S23-A	0.08	1.90E+00	29	12-93 (107)	47-124 (145)
AF-P26754-F1-model_v4	Replication factor A protein 2	0.08	6.91E+00	29	13-98 (107)	70-159 (273)
AF-P20459-F1-model_v4	Eukaryotic translation initiation factor 2 subunit alpha	0.08	3.42E+00	29	12-92 (107)	18-86 (304)
AF-P38859-F1-model_v4	DNA replication ATP-dependent helicase/nuclease DNA2	0.08	5.16E+00	29	3-82 (107)	397-500 (1522)
AF-P0C0X0-F1-model_v4	40S ribosomal protein S28-B	0.07	9.27E+00	27	10-69 (107)	4-61 (67)
AF-P20436-F1-model_v4	DNA-directed RNA polymerases I, II, and III subunit RPABC3	0.06	8.74E+00	25	11-72 (107)	5-69 (146)
AF-P29366-F1-model_v4	Bud emergence protein 1	0.03	6.52E+00	21	1-95 (107)	145-208 (551)

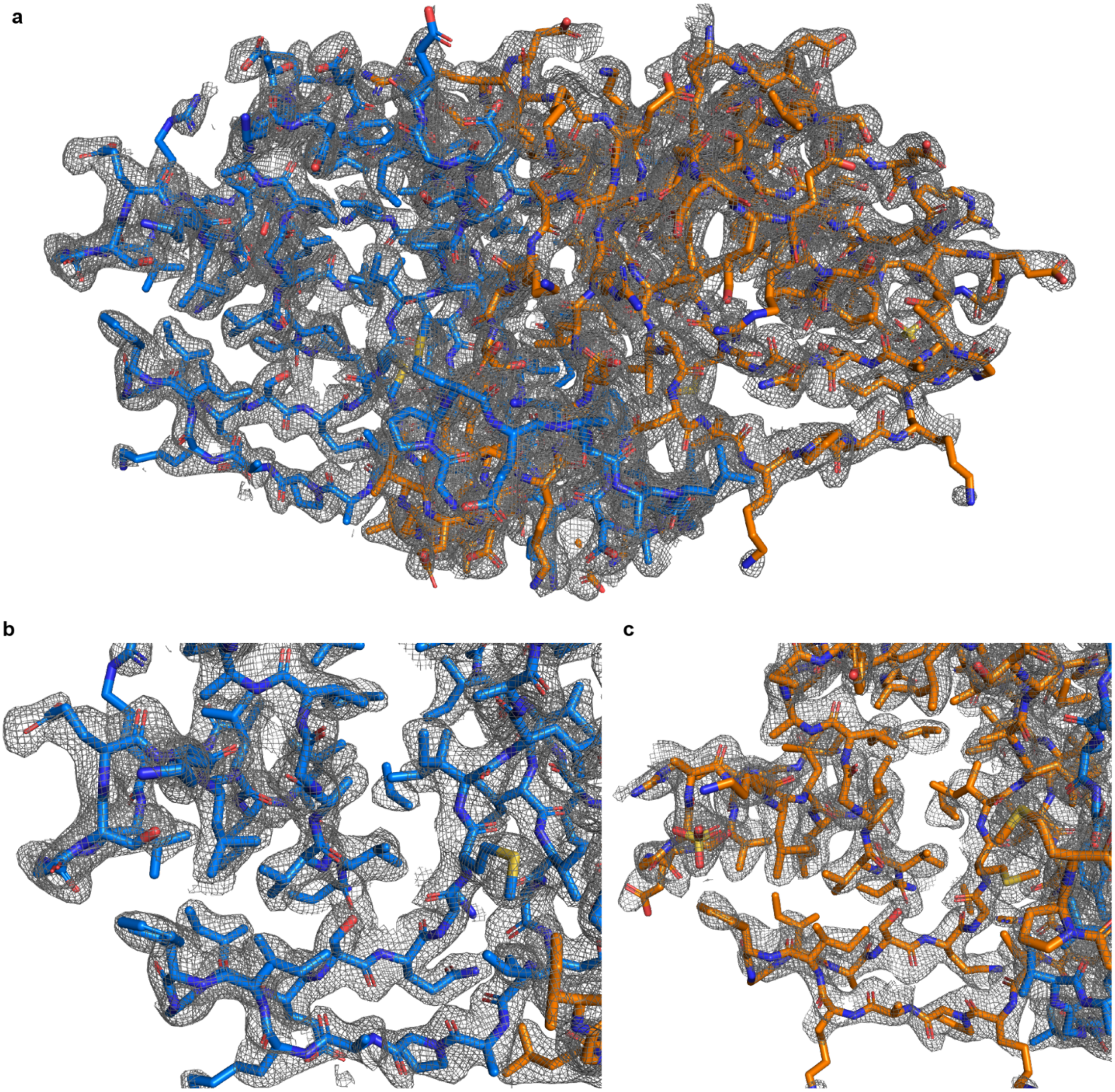

Supplementary Fig. 1 | Composite simulated anneal-omit 2|F_o|-|F_c| electron density for unbound A. aeolicus Trbp111 contoured at 1.0 σ superimposed with the final model. a, Full Trbp111 dimer. **b**, Zoomed-in view of the L_{12} and L_{45} loop region in chain A. **c**, Zoomed-in view of the L_{12} and L_{45} loop region in chain B. All figures rendered with MacPyMOL.

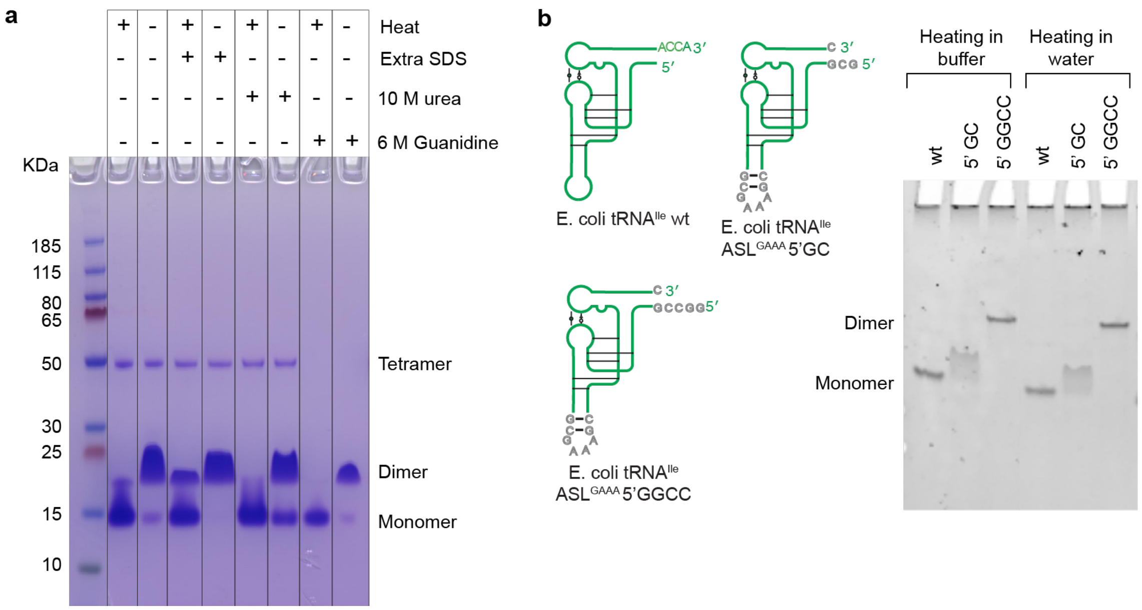

Supplementary Fig. 2 | Electrophoresis analysis of Trbp111 and tRNA^{Ile} constructs. a, SDS-PAGE analysis of Trbp111. All samples were initially prepared in a loading buffer containing 0.62 % w/v SDS. Before electrophoresis, samples were further treated with either additional 1% SDS (1.62 % total), 10 M urea, or 6 M guanidine with or without heat. Samples were then analyzed on a 4-12% Bis-Tris SDS-PAGE gel in MES buffer. **b**, Non-denaturing PAGE analysis of refolded *E. coli* tRNA^{Ile} constructs. Heating in buffer: 20 μM tRNA was heated at 90 ºC in 25 mM Tris-HCl pH 7.4, 150 mM NaCl for 3 min, snap-cooled in an ice bath for 3 min, then 2 mM $MgCl_2$ was added. Heating in water: 22 μM tRNA was heated at 90 ºC in water for 3 min, snap-cooled in an ice bath for 3 min, then buffer was added to a final concentration of 25 mM Tris-HCl pH 7.4, 150 mM NaCl, 2 mM $MgCl_2$. The 5′ GGCC construct forms a strong dimer in both cases, while the 5′ GC construct exhibits weaker dimerization.

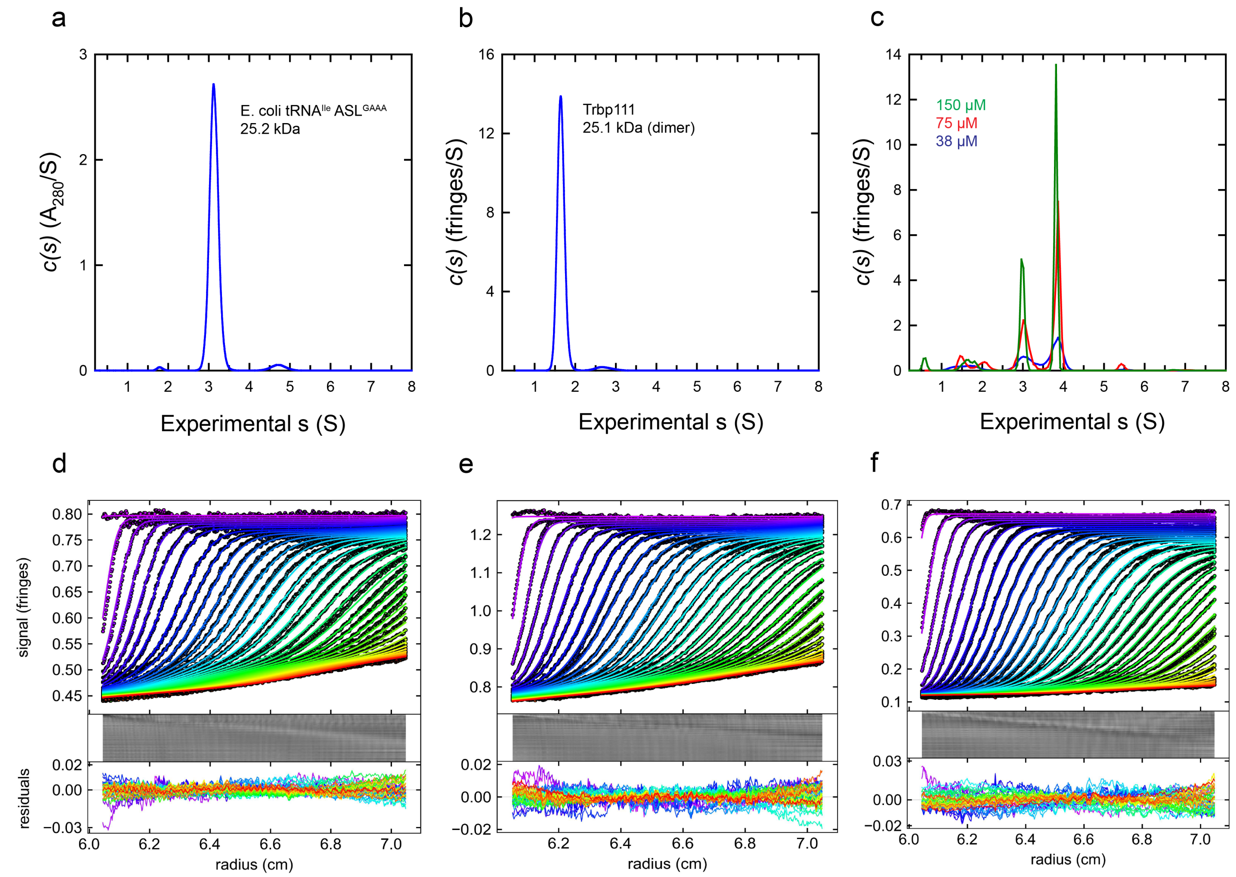

Supplementary Fig. 3 | Sedimentation velocity AUC analyses of tRNA^{Ile}, Trbp111, and their complexes. a, Absorbance c(*s*) distribution for 3.1 μM tRNA^{Ile}-ASL^{GAAA} loaded in a 12 mm pathlength cell showing a species at 3.13 S with an estimated molar mass of 25.2 kDa. **b,** Interference c(*s*) distribution for 74 μM Trbp111 loaded in a 12 mm pathlength cell showing a species at 1.65 S with an estimated molar mass of 25.1 kDa indicative of a Trbp111 dimer. **c**, Interference c(*s*) distributions for equimolar mixtures of 38 μM (blue), 75 μM (red), and 150 μM (green) tRNA^{Ile}-ASL^{GAAA} and Trbp111 (concentration in monomer subunits). The sample at the highest concentration was studied in a 1.5 mm pathlength cell (Nanolytics GmbH), whereas the other samples were studied in 3 mm pathlength cells. Note the predominant presence of a complex at 3.83 S indicative of a 1:2 tRNA:Trbp111 stoichiometry, along with free tRNA at 3.04 S and free Trbp111. Based on the complex structure, HYDROPRO modeling returns experimental sedimentation coefficients of 1.68 S and 3.03 S for the Trbp111 dimer and free tRNA. Sedimentation coefficients of 3.34 S and 4.35 S are determined for the 1:2 and 2:2 tRNA:Trbp111 complexes, respectively. **d**, **e**, and **f** show interference sedimentation velocity scans for samples containing 5.1 μM Trbp111 dimer and 11 μM (**d**), 16 μM (**e**), and 21 μM (**f**) tRNA^{Ile}-ASL^{GAAA} in 3 mm pathlength cells. Data were fit using the Lamm equations describing an A + B + B = (AB) + B = ABB model with two symmetric sites and macroscopic K in SEDPHAT, where A represents the Trbp111 dimer and B the tRNA. The model returns a K_d of 6.3 μM (68% confidence interval = 5.5 – 7.6 μM) for the first binding event, and a K_d of 215 μM (68% confidence interval = 150 – 1490 μM) for the second binding event, indicative of a high negative co-operativity. Sedimentation coefficients for all species were fixed in the analysis. S-values for the Trbp111 dimer, tRNA, and 1:2 complex were fixed to experimentally determined values. The value determined in

HYDROPRO was used for the 2:2 complex. The GUSSI[3] plots show the raw data and best-fits in the top panels, with the residuals in bitmap and plot forms below. For clarity only every third scan and every third data point are shown. All data were collected at 10°C.

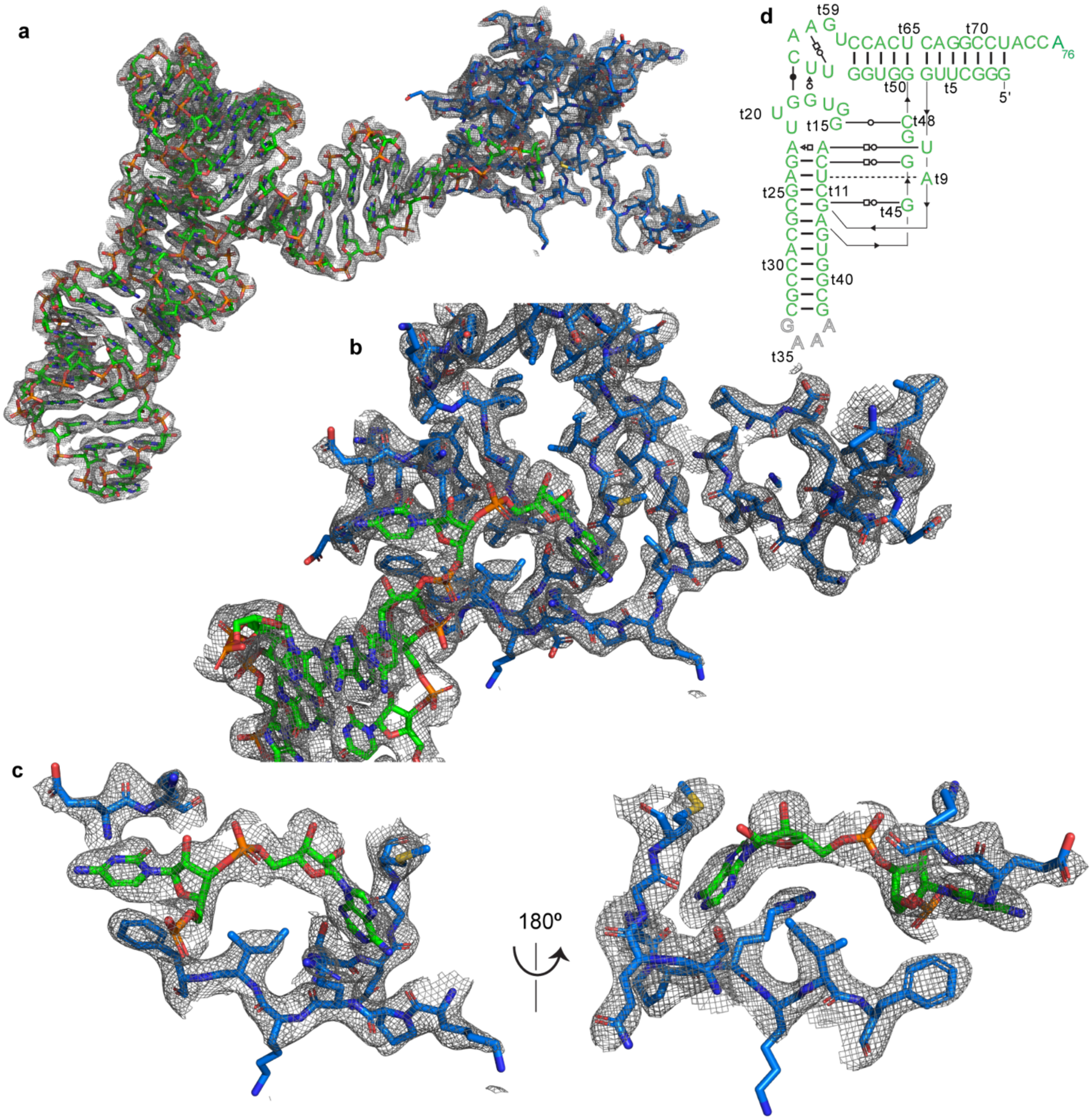

Supplementary Fig. 4 | Composite simulated anneal-omit $2|F_o|-|F_c|$ electron density for the Trbp111-tRNA^{Ile} complex contoured at 1.0 σ superimposed to the final refined model. a, Trbp111 chain A complexed with tRNA chain C. **b**, Zoomed-in view of the interface. **c**, Only interacting residues are shown. **d**, Sequence and secondary structure of the *E. coli* tRNA^{Ile} ASL^{GAAA} used for crystallization. Residues in gray were engineered to facilitate crystallization. All figures rendered with MacPyMOL.

a

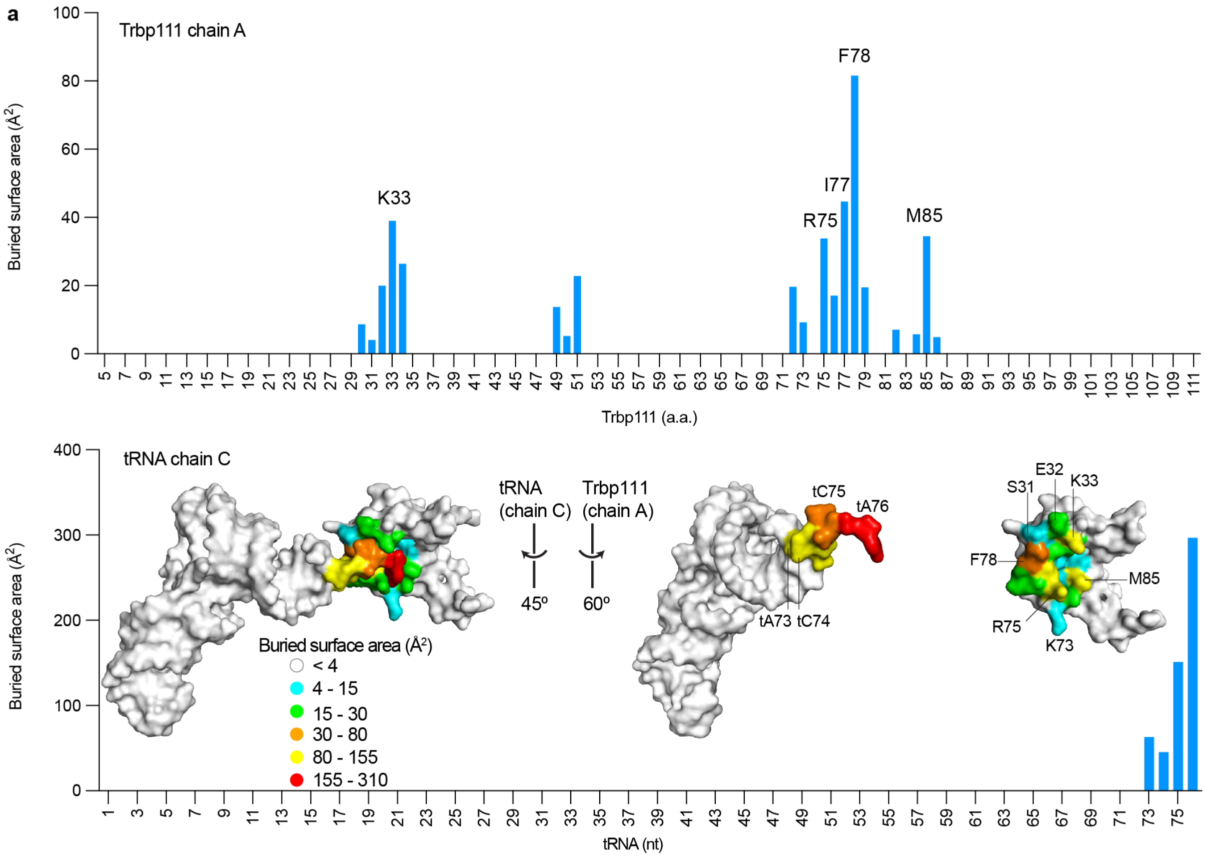

Trbp111 chain A
Buried surface area (Å²)
Trbp111 (a.a.)
K33
R75
I77
F78
M85
tRNA chain C
tRNA (nt)
tRNA (chain C)
Trbp111 (chain A)
45º
60º
tC75
tA76
tA73
tC74
S31
E32
K33
F78
M85
R75
K73
Buried surface area (Å²)
< 4
4 - 15
15 - 30
30 - 80
80 - 155
155 - 310

b

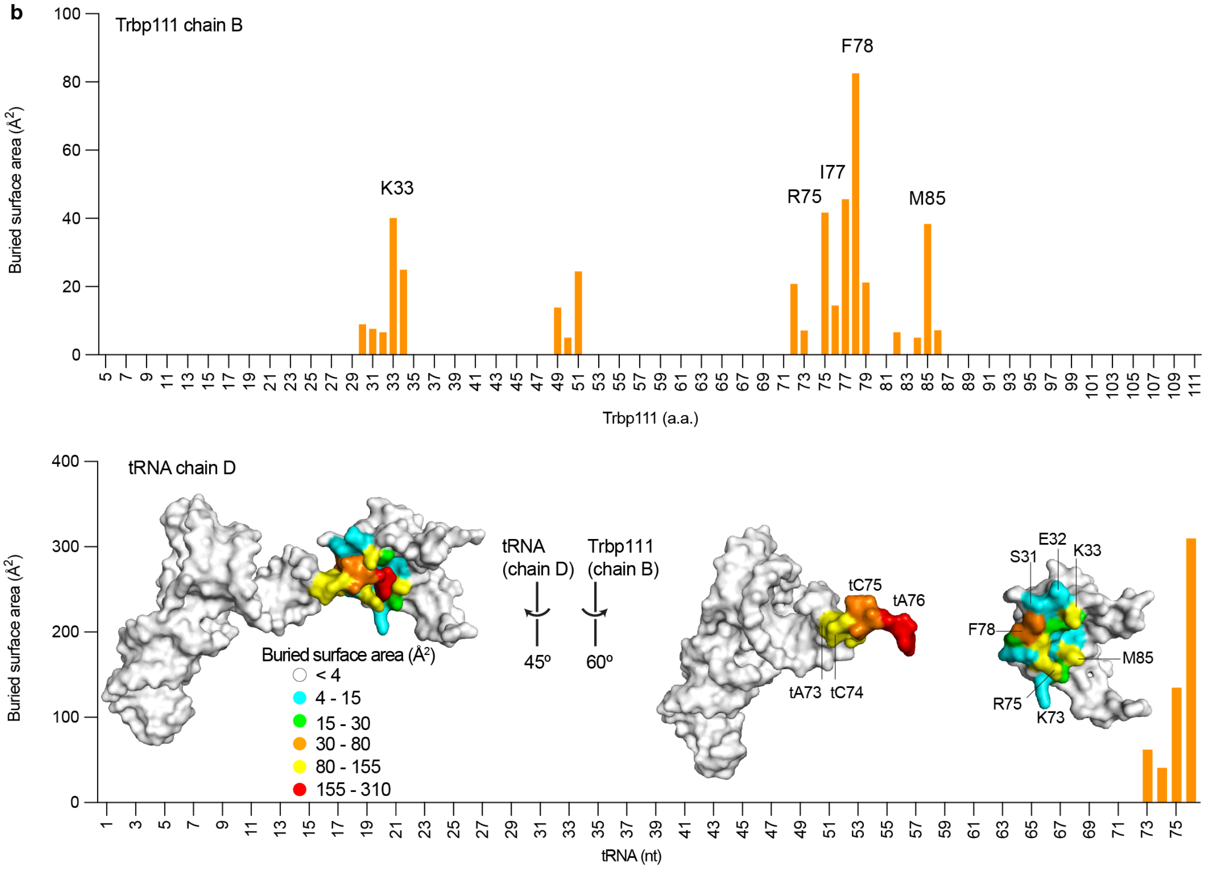

Trbp111 chain B
Buried surface area (Å²)
Trbp111 (a.a.)
K33
R75
I77
F78
M85
tRNA chain D
tRNA (nt)
tRNA (chain D)
Trbp111 (chain B)
45º
60º
tC75
tA76
tA73
tC74
S31
E32
K33
F78
M85
R75
K73
Buried surface area (Å²)
< 4
4 - 15
15 - 30
30 - 80
80 - 155
155 - 310

Supplementary Fig. 5 | Analysis of solvent-accessible surface area buried by the Trbp111-tRNA interface. a, Surface area buried per residue by the interaction between Trbp111 chain A (top) and tRNA chain C (bottom). Insert: solvent-accessible surface colored by area buried on chains A and C from white (no burial) to red (155 – 310 Å^2 per residue). **b**, Surface area buried per residue by the interaction between Trbp111 chain B (top) and tRNA chain D (bottom). Insert: solvent-accessible surface colored by area buried on chains B and D from white (no burial) to red (155 – 310 Å^2 per residue). Interfaces were analyzed by StrucTools (https://hpcnihapps.cit.nih.gov/structbio/). Plots were generated by Excel. Structural images rendered by MacPyMOL.

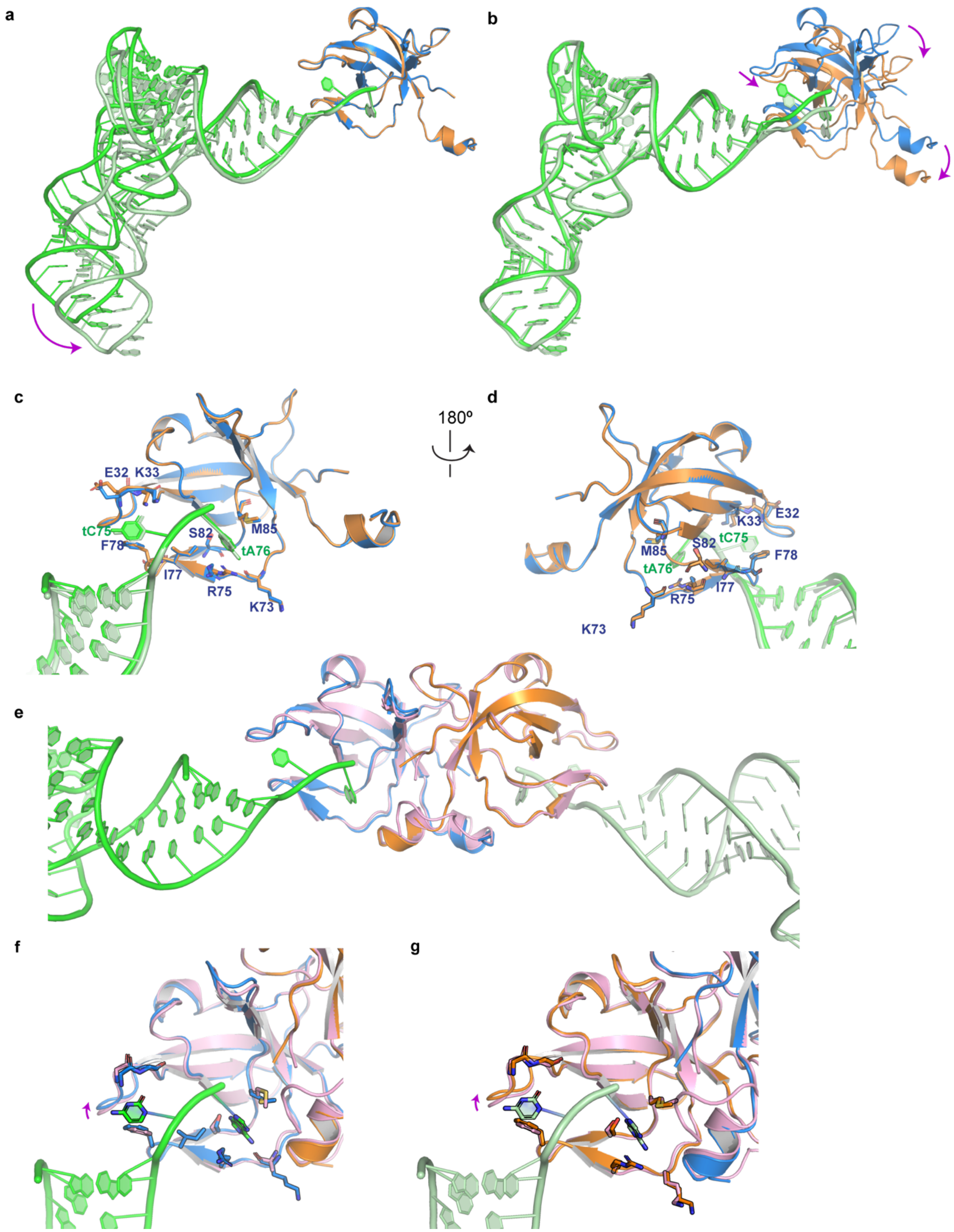

a
b
c
E32 K33
tC75
F78
S82
M85
tA76
I77
R75
K73
180º
d
E32
K33
M85
S82 tC75
F78
tA76
I77
R75
K73
e
f
g

Supplementary Fig. 6 | Structural comparison between the two observed tRNA-binding interfaces and with free Trbp111. a, b, Overlay of two crystallographically observed Trbp111-tRNA complexes aligned on Trbp111 (**a**, RMSD ~ 0.2 Å), or on tRNA (**b**, RMSD ~ 0.4 Å). Magenta arrows indicate chain movements. **c, d**, Two zoomed-in views of (**a**) showing the conservation of the binding interface. **e**, Overlay of unbound Trbp111 (pink) to Trbp111 in the complex structure (blue and orange). **f, g**, Zoomed-in views of (**e**), showing the chain A (Trbp111) – chain C (tRNA) interface (**f**) and chain B (Trbp111) – chain D (tRNA) interface (**g**). Magenta arrows indicate movement of Trbp111 loops upon tRNA binding.

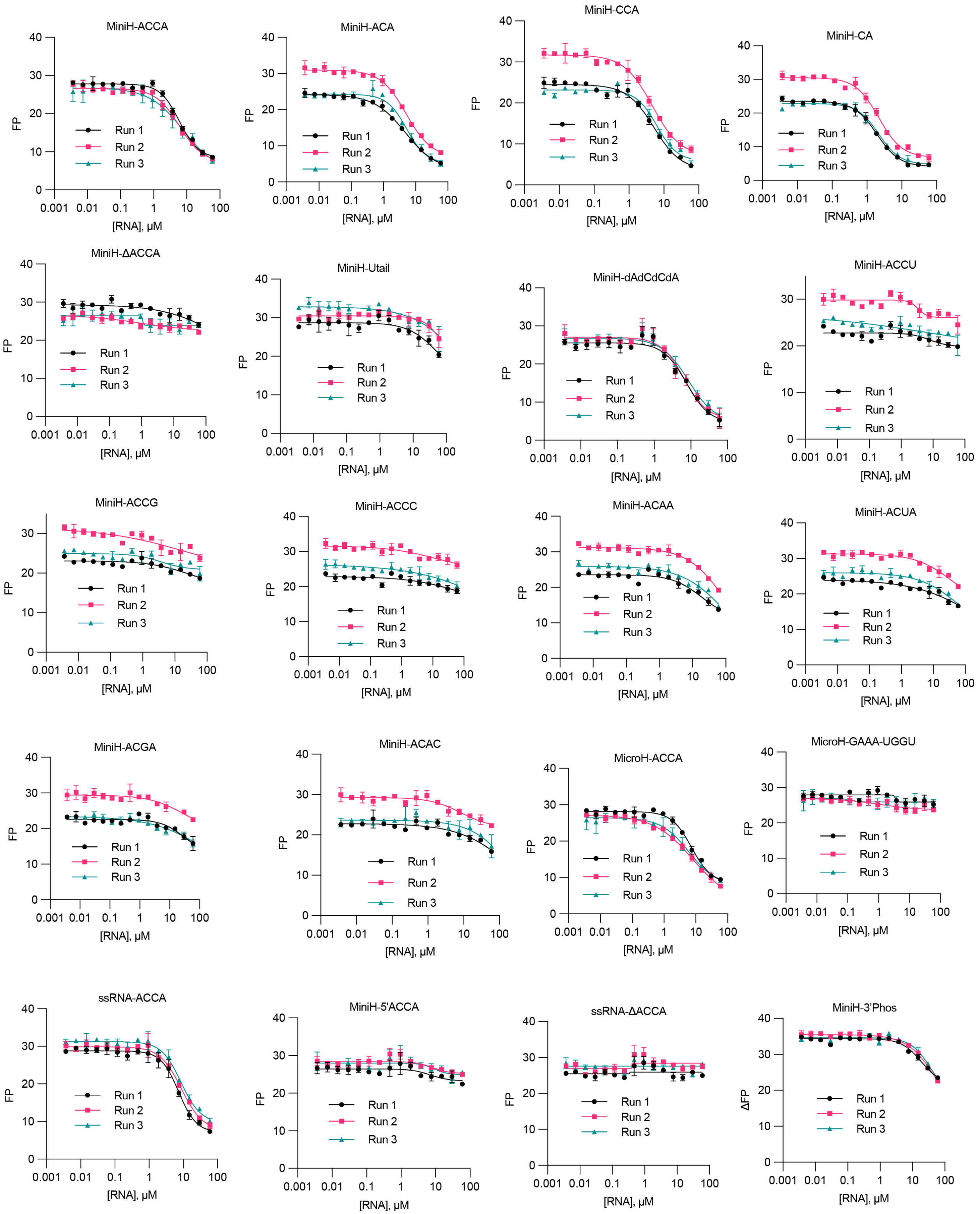

Supplementary Fig. 7 | Competition fluorescence polarization binding assays of Trbp111 to minihelices (MiniH), microhelices (MicroH) and ssRNA. Secondary structures and sequences are indicated in Figure 3.

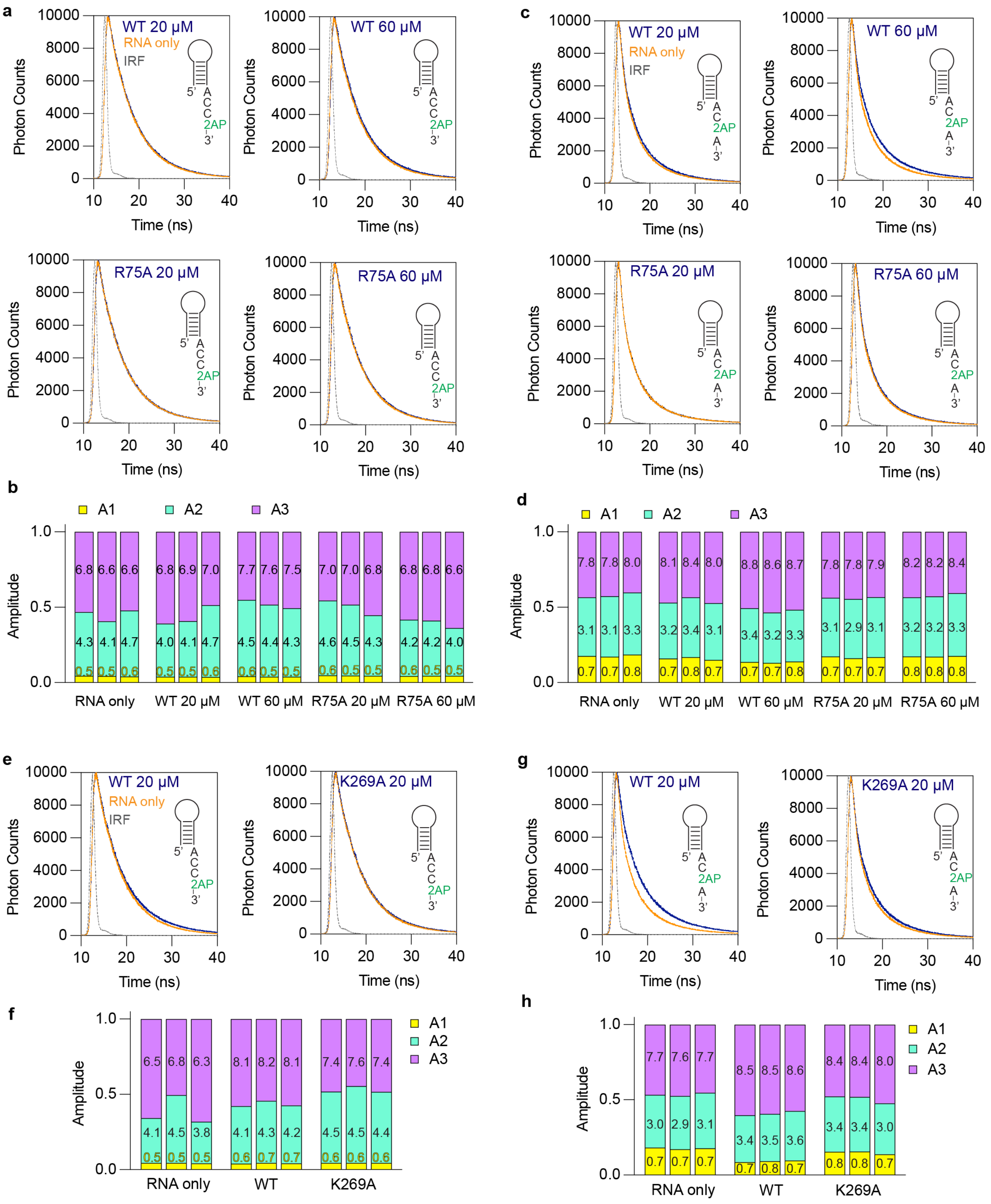

a
WT 20 μM
RNA only
IRF
Photon Counts
Time (ns)
WT 60 μM
R75A 20 μM
R75A 60 μM
5'
A
C
C
2AP
3'
b
A1
A2
A3
Amplitude
RNA only
WT 20 μM
WT 60 μM
R75A 20 μM
R75A 60 μM
6.8 6.6 6.6 6.8 6.9 7.0 7.7 7.6 7.5 7.0 7.0 6.8 6.8 6.8 6.6
4.3 4.1 4.7 4.0 4.1 4.7 4.5 4.4 4.3 4.6 4.5 4.3 4.2 4.2 4.0
0.5 0.5 0.6 0.5 0.5 0.6 0.6 0.5 0.5 0.6 0.5 0.5 0.6 0.5 0.5
c
WT 20 μM
RNA only
IRF
WT 60 μM
R75A 20 μM
R75A 60 μM
5'
A
C
2AP
A
3'
d
A1
A2
A3
7.8 7.8 8.0 8.1 8.4 8.0 8.8 8.6 8.7 7.8 7.8 7.9 8.2 8.2 8.4
3.1 3.1 3.3 3.2 3.4 3.1 3.4 3.2 3.3 3.1 2.9 3.1 3.2 3.2 3.3
0.7 0.7 0.8 0.7 0.8 0.7 0.7 0.7 0.8 0.7 0.7 0.7 0.8 0.8 0.8
RNA only
WT 20 μM
WT 60 μM
R75A 20 μM
R75A 60 μM
e
WT 20 μM
RNA only
IRF
K269A 20 μM
f
A1
A2
A3
6.5 6.8 6.3 8.1 8.2 8.1 7.4 7.6 7.4
4.1 4.5 3.8 4.1 4.3 4.2 4.5 4.5 4.4
0.5 0.5 0.5 0.6 0.7 0.7 0.6 0.6 0.6
RNA only
WT
K269A
g
WT 20 μM
K269A 20 μM
h
A1
A2
A3
7.7 7.6 7.7 8.5 8.5 8.6 8.4 8.4 8.0
3.0 2.9 3.1 3.4 3.5 3.6 3.4 3.4 3.0
0.7 0.7 0.7 0.7 0.8 0.7 0.8 0.8 0.7
RNA only
WT
K269A

Supplementary Fig. 8 | Effects of Trbp111 and Arc1p binding on the fluorescence lifetimes of 2AP incorporated at or near the tRNA 3′ end. a-b, Time-resolved 2AP fluorescence decay time traces (**a**) and individual lifetime deconvolutions (**b**) of a tRNA minihelix carrying 2AP at the terminal position tA76 (MiniH-ACC-2AP), in the absence (orange lines) or presence (dark blue lines) of WT or R75A Trbp111. IRF: instrument response function (gray lines). **c**-**d**, same analysis as in (**a**-**b)** but using a minihelix carrying 2AP at the penultimate position tC75 (MiniH-AC-2AP-A). **e**-**f**, Time-resolved 2AP fluorescence decay time traces (**e**) and individual lifetime deconvolutions (**f**) of MiniH-ACC-2AP, in the absence (orange lines) or presence (dark blue lines) of WT or K269A Arc1pΔN. **g**-**h**, same analysis as in (**e**-**f)** but using MiniH-AC-2AP-A. Three biologically independent replicate measurements are shown as adjacent stacked columns. Lifetime values are indicated in nanoseconds. A1, A2, and A3 are the relative amplitudes of the deconvoluted short (yellow), medium (cyan), and long (purple) individual photon-emitting species, respectively. The amplitude-weighted averaged lifetimes τ_{avg} are summarized in Figs. 3h, 3j, 6b, and 6d.

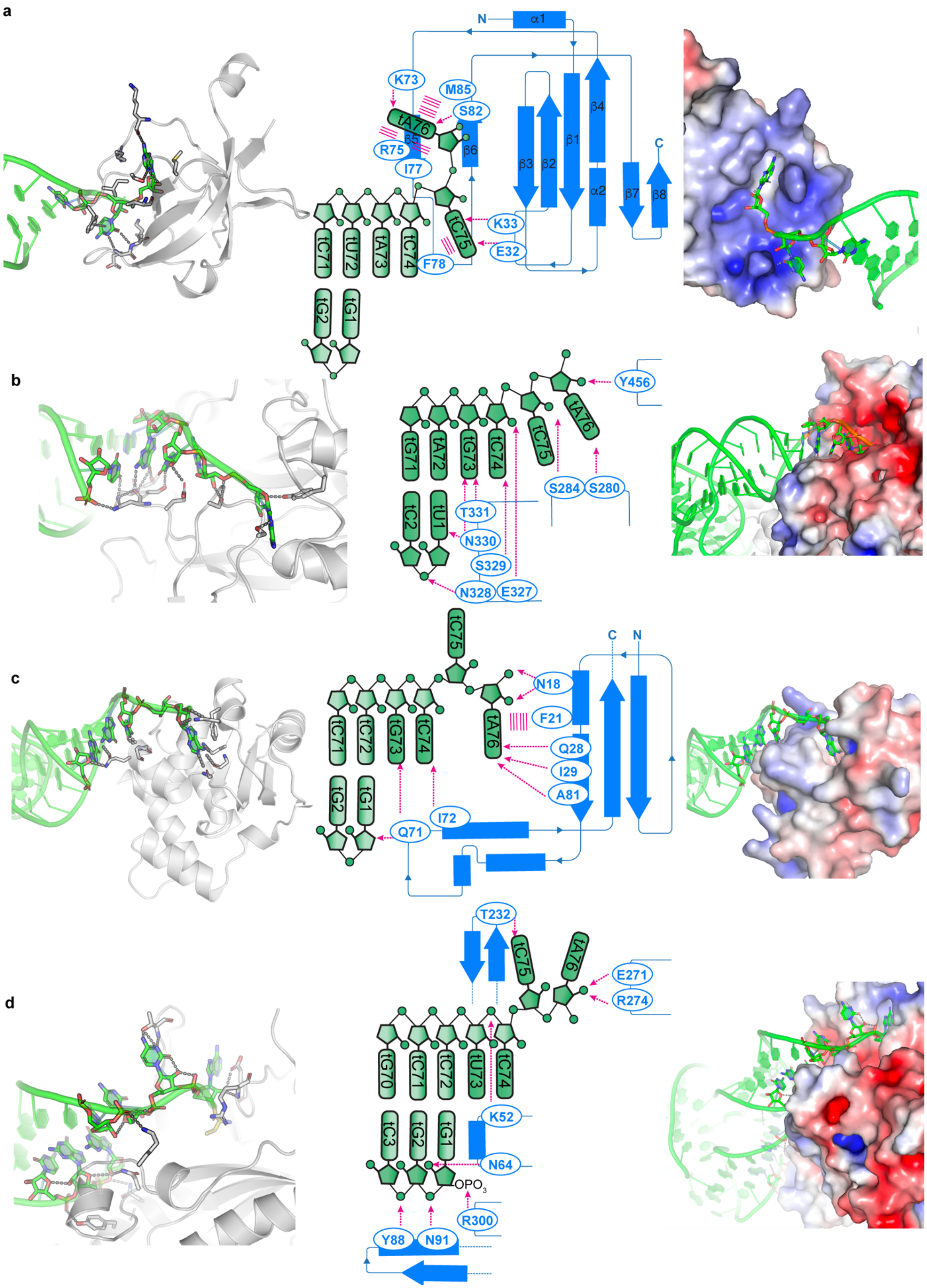
a
N
α1
C
β1
β2
β3
β4
β5
β6
β7
β8
α2
K73
M85
S82
tA76
R75
I77
K33
E32
F78
tC75
tC71
tU72
tA73
tC74
tG2
tG1
b
Y456
tA76
tC75
tG71
tA72
tG73
tC74
tC2
tU1
S284
S280
T331
N330
S329
N328
E327
c
tC75
C
N
N18
F21
Q28
I29
A81
tA76
tC71
tC72
tG73
tC74
tG2
tG1
I72
Q71
d
T232
tC75
tA76
E271
R274
tG70
tC71
tC72
tU73
tC74
tC3
tG2
tG1
K52
N64
OPO3
R300
Y88
N91

Supplementary Fig. 9 | Comparison of the tRNA 3′ end recognition mechanisms employed by Trbp111, aaRSs, KEOPS complex, and EF-Tu. a-d, Co-crystal structures (left), cartoon schematics (center), and electrostatic potential surfaces (right) of the Trbp111-tRNA complex (**a**), yeast aspartyl-tRNA synthetase (AspRS)-tRNA^{Asp} complex (**b**, PDB 1ASY), tRNA complex with the CGI121 subunit of the KEOPS tRNA modifying complex (**c**, PDB 7KJT), and *Thermus aquaticus* EF-Tu-tRNA^{Cys} complex (**d**, PDB 1B23)[4-6]. Dashed arrows indicate hydrogen bonds; stacked parallel lines indicate stacking interactions.

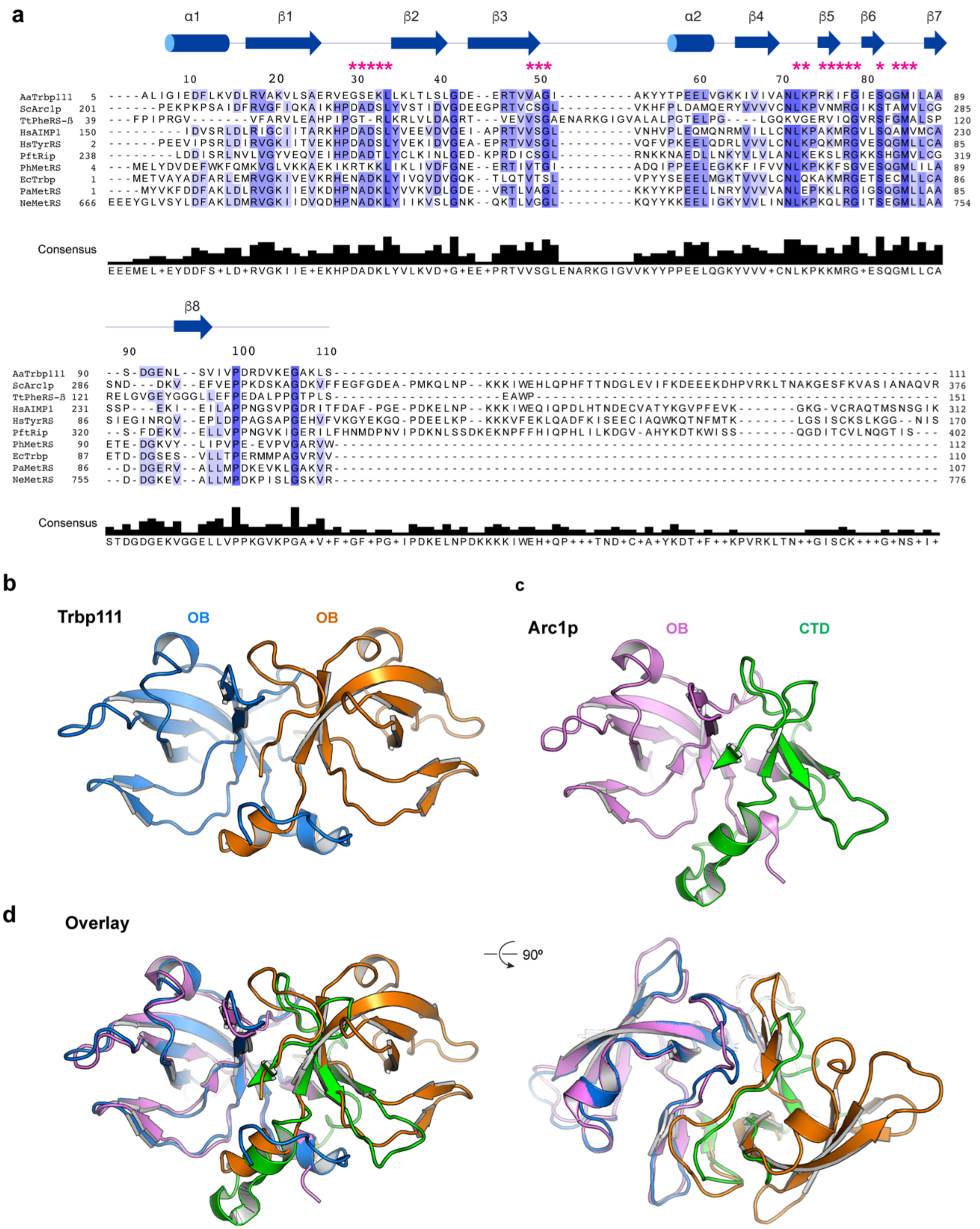

Supplementary Fig. 10 | Sequence and structural alignments of selected Trbp/EMAPII-like domains. a, Sequence alignment with Trbp111 secondary structural features. Asterisks indicate interfacial residues identified by PDBePISA interface analysis of the co-crystal structure. **b-d**, Structural comparison of *S. cerevisiae* Arc1p-OB-CTD (**b**, a.a. 201-376, PDB: 4R1J[7]), free Trbp111 (**c**), and two views of their structural overlay (**d**).

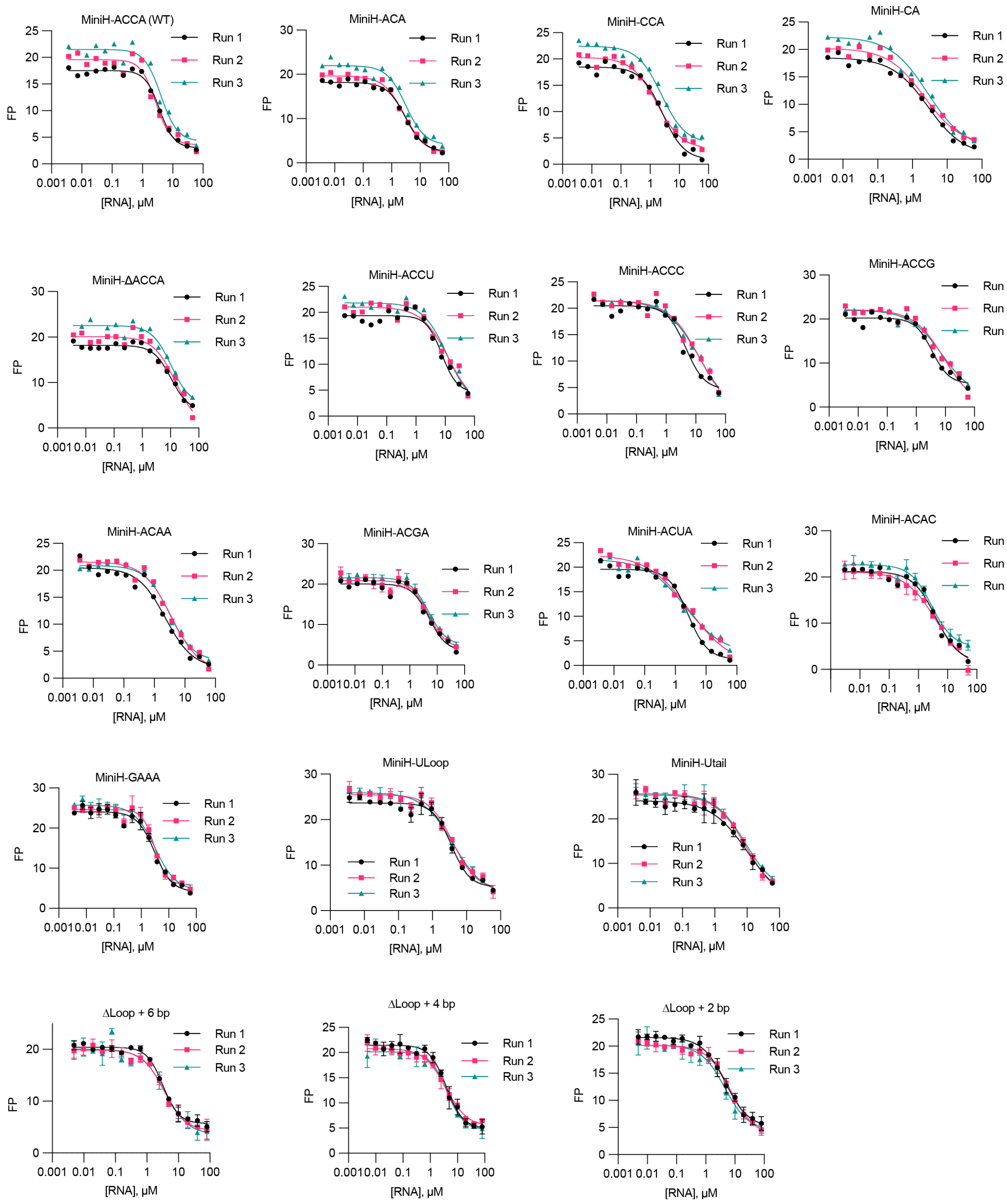

Supplementary Fig. 11 | Competition fluorescence polarization binding assays of Arc1pΔN to minihelices (MiniH). Secondary structures and sequences are indicated in Figure 6.

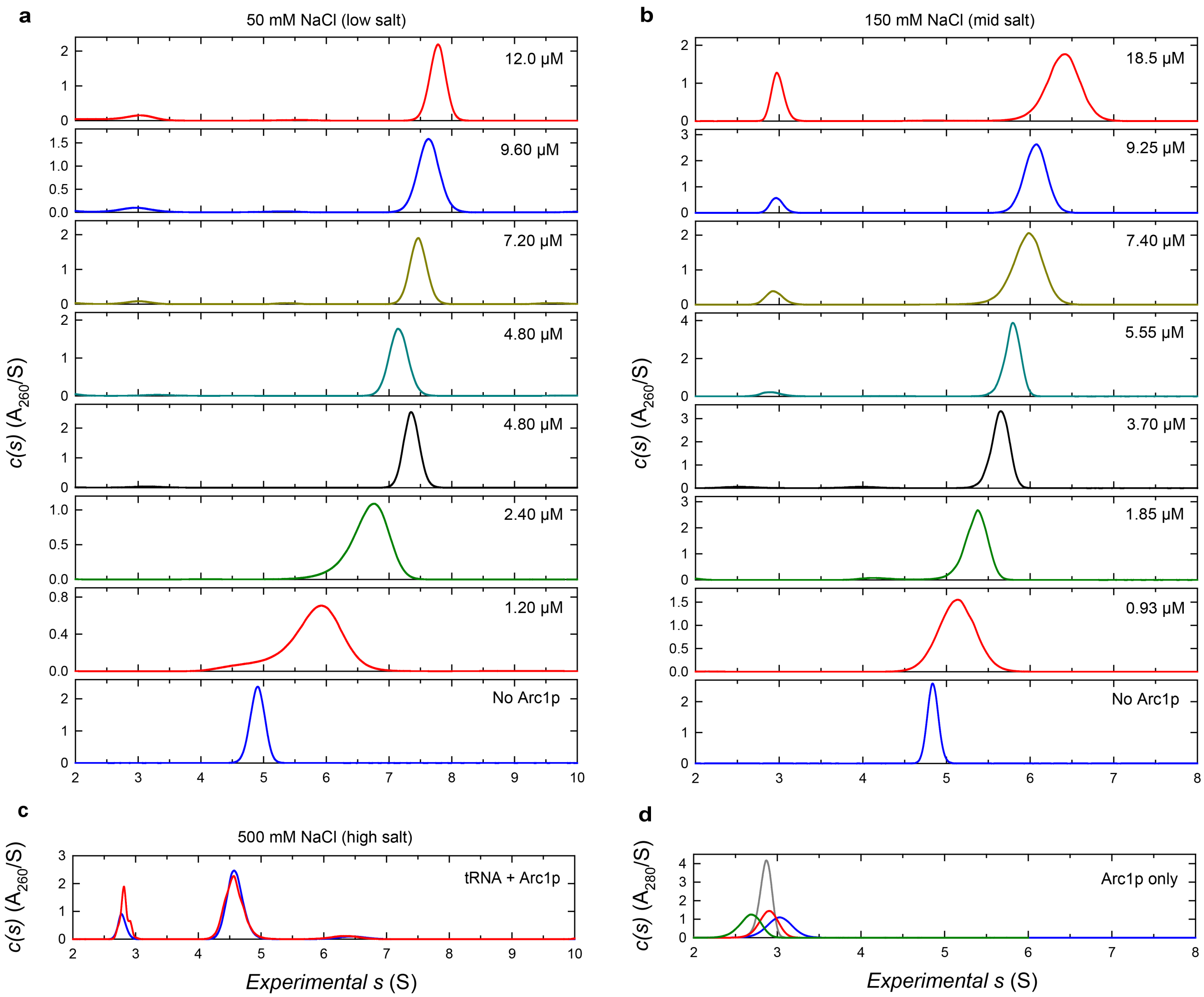

Supplementary Fig. 12 | Sedimentation velocity AUC analyses of Arc1p and its complexes with tRNA. a, Absorbance (260 nm) c(s) distributions for 0.7 μM $tRNA^{Tyr}$ in low salt buffer (50 mM NaCl) along with increasing amounts of added Arc1p (indicated). Without added protein, $tRNA^{Tyr}$ sediments at 4.91 S with an estimated molar mass of 28.1 kDa. Increasing concentrations of Arc1p, as indicated, leads to the formation of both 1:1 and 1:2 tRNA:Arc1p complexes. Data suggest a high-affinity interaction and a weaker, less specific interaction. Evidence for free protein (~ 3.0 S) is noted at higher concentrations. **b**, Absorbance (260 nm) c(s) distributions for 1.08 μM $tRNA^{Tyr}$ in mid-salt buffer (150 mM NaCl) and increasing Arc1p. Free $tRNA^{Tyr}$ sediments at 4.87 S with an estimated molar mass of 29.3 kDa. The addition of Arc1p leads to the formation of both 1:1 and 1:2 tRNA:Arc1p complexes with diminished affinities compared to data collected in 50 mM NaCl (**a**). **c**, Absorbance (260 nm) c(s) distributions for 1.0 μM $tRNA^{Tyr}$ mixed with 10 μM (blue) or 20 μM (red) Arc1p in high salt buffer (500 mM NaCl). Note the absence of complex formation. The species between 6 and 7 S represents traces of a tRNA dimer impurity. **d**, Absorbance (280 nm) c(s) distributions for 12 μM Arc1p in low (blue), mid (red), or high (green) salt buffers using 12 mm pathlength cells. Arc1p was also studied in mid-salt buffer and a 1.5 mM pathlength cell at 190 μM (grey). In all cases, data indicate an Arc1p monomer. All data were collected at 25°C.

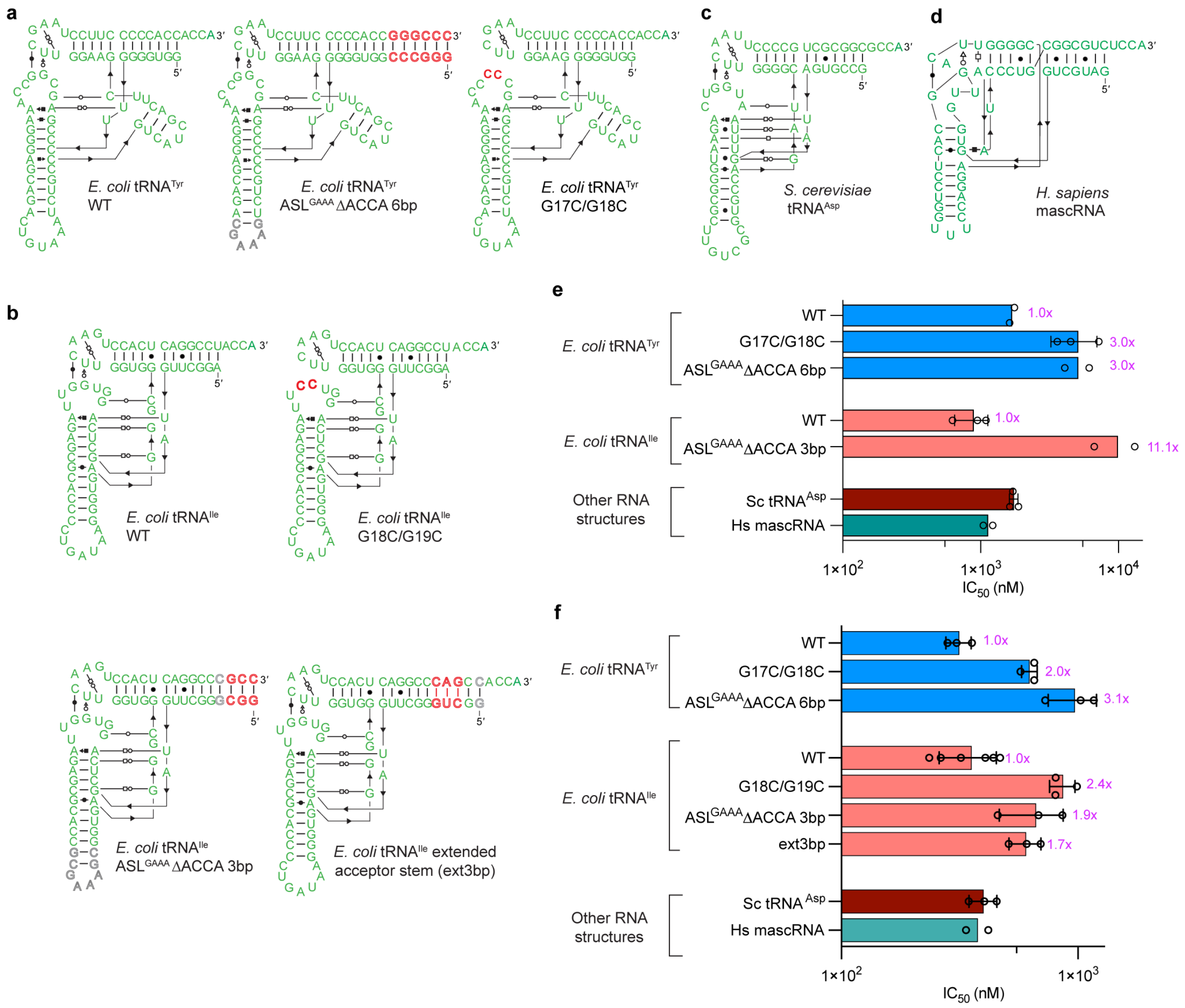

Supplementary Fig. 13 | Arc1p competition fluorescence polarization assay with full-length tRNA and tRNA-like structures. a-d, Sequences and secondary structures of RNA constructs used, including *E. coli* $tRNA^{Tyr}$ (**a**), *E. coli* $tRNA^{Ile}$ (**b**), *S. cerevisiae* $tRNA^{Asp}$ (**c**), and *H. sapiens* mascRNA (**d**). **e**, IC_{50} values derived from full-length Arc1p FP competition assays. Data are mean ± s.d. from n biologically independent replicates. n = 2 for WT *E. coli* $tRNA^{Tyr}$, ASL^{GAAA}/ΔACCA 6bp *E. coli* $tRNA^{Tyr}$, human mascRNA; n = 3 for G17C/G18C *E. coli* $tRNA^{Tyr}$, WT *E. coli* $tRNA^{Ile}$, *S. cerevisiae* $tRNA^{Asp}$. **f**, IC_{50} values derived from Arc1pΔN competition assays. Data are mean ± s.d. from n biologically independent replicates. n = 3 for WT, G17C/G18C, and ASL^{GAAA}/ΔACCA 6bp *E. coli* $tRNA^{Tyr}$, and G18C/G19C, ASL^{GAAA}/ΔACCA 3bp, and ext3bp *E. coli* $tRNA^{Ile}$, and *S. cerevisiae* $tRNA^{Asp}$; n = 4 for WT *E. coli* $tRNA^{Ile}$; n = 2 for human mascRNA.

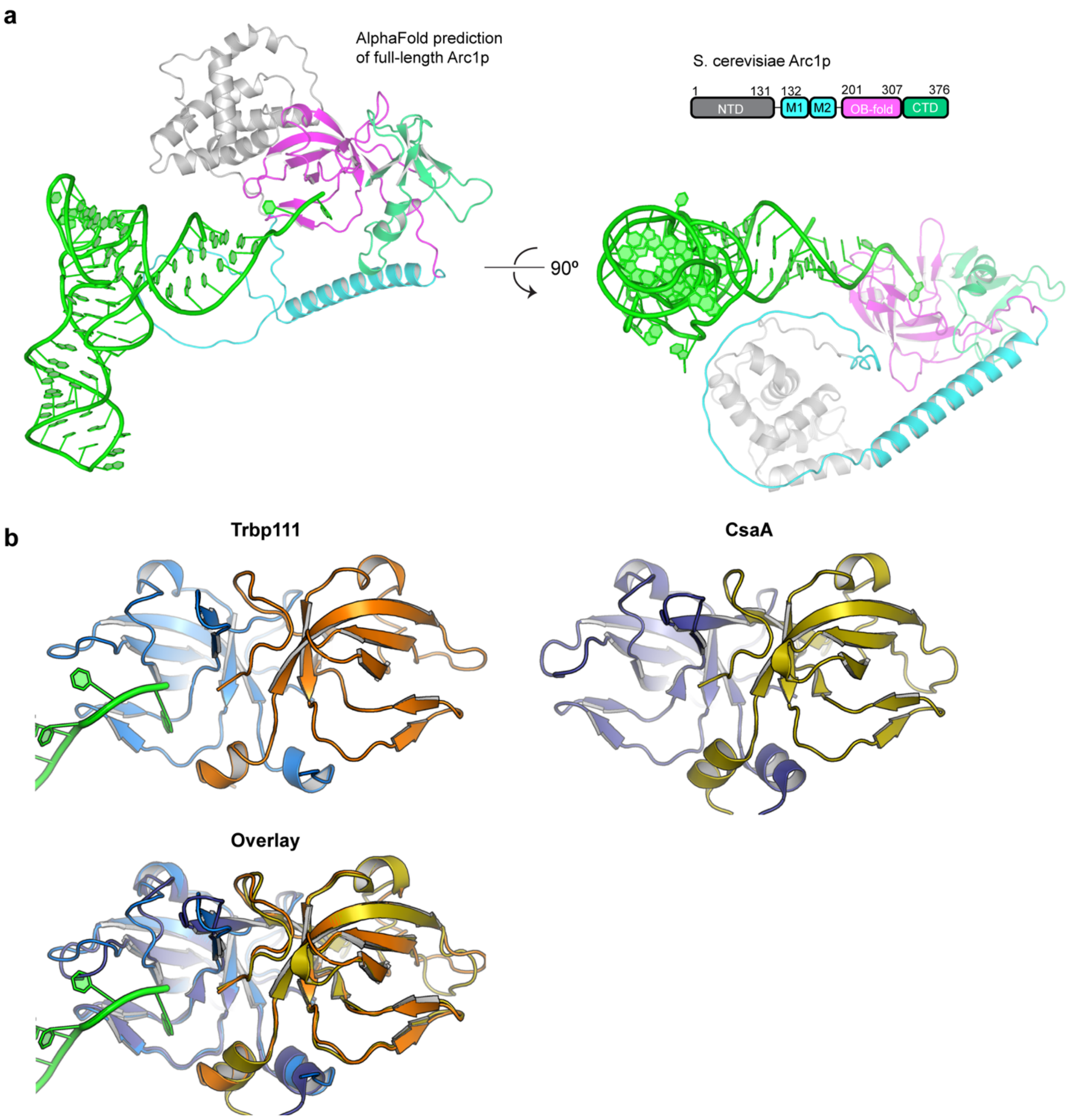

Supplementary Fig. 14 | Structural comparison with AlphaFold model of full-length Arc1p and CsaA. a, Structural model of full-length Arc1p bound to tRNA, generated by overlaying the AlphaFold2 model of Arc1p (AF- C8Z8E3-F1)[8], as is, with the Trbp111-tRNA co-crystal structure by their homologous OB folds, without fitting. **b**, Structures of Trbp111-tRNA complex (left), *Thermus thermophilus* CsaA (PDB:1GD7, right)[9], and their overlay (lower left).

Supplementary References

1. van Kempen, M. et al. Fast and accurate protein structure search with Foldseek. *Nat Biotechnol* **42**, 243-246 (2024).
2. Swairjo, M.A., Morales, A.J., Wang, C.C., Ortiz, A.R. & Schimmel, P. Crystal structure of trbp111: a structure-specific tRNA-binding protein. *EMBO J* **19**, 6287-98 (2000).
3. Brautigam, C.A. Calculations and Publication-Quality Illustrations for Analytical Ultracentrifugation Data. *Methods Enzymol* **562**, 109-33 (2015).
4. Ruff, M. et al. Class II Aminoacyl Transfer RNA Synthetases: Crystal Structure of Yeast Aspartyl-tRNA Synthetase Complexed with tRNAAsp. *Science* **252**, 1682-1689 (1991).
5. Beenstock, J. et al. A substrate binding model for the KEOPS tRNA modifying complex. *Nature Communications* **11**, 6233 (2020).
6. Nissen, P., Thirup, S., Kjeldgaard, M. & Nyborg, J. The crystal structure of Cys-tRNACys–EF-Tu–GDPNP reveals general and specific features in the ternary complex and in tRNA. *Structure* **7**, 143-156 (1999).
7. Giessen, T.W. et al. A synthetic adenylation-domain-based tRNA-aminoacylation catalyst. *Angew Chem Int Ed Engl* **54**, 2492-6 (2015).
8. Jumper, J. et al. Highly accurate protein structure prediction with AlphaFold. *Nature* **596**, 583-589 (2021).
9. Kawaguchi, S. et al. The crystal structure of the ttCsaA protein: an export-related chaperone from Thermus thermophilus. *Embo j* **20**, 562-9 (2001).
